# Gain Enhancement of the Optical Waveguide Amplifier Based on NaYF_4_/NaLuF_4_: Yb, Er NPs-PMMA Integrated with a Si_3_N_4_ Slot

**DOI:** 10.3390/nano12172937

**Published:** 2022-08-25

**Authors:** Xiao Liu, Meiling Zhang, Guijun Hu

**Affiliations:** Department of Communication Engineering, Jilin University, Changchun 130012, China

**Keywords:** Si_3_N_4_ slot waveguide, optical waveguide amplifier, nanocrystals

## Abstract

A Si_3_N_4_ slot waveguide has the ability to confine light tightly in the slot, shows weak absorption of 980 nm pump light, and has lower transmission loss compared to a Si slot. Hence, the optical waveguide amplifier based on Er^3+^ and Yb^3+^codoped was proposed to be integrated with a Si_3_N_4_ slot to increase the gain. The core-shell NaYF_4_/NaLuF_4_: 20%Yb^3+^, 2%Er^3+^ nanocrystals-polymeric methyl methacrylate covalent linking nanocomposites were synthesized and filled into the slot as gain medium. The concentrations of Er^3+^ and Yb^3+^ were increased compared with traditional physical doping methods. High-efficiency emission at 1.53 μm was achieved under 980 nm laser excitation. The slot waveguide was accurately designed using the semivector finite difference method in combination with the maximum confinement factors and the minimum effective mode area. The optimum width of the slot was 200 nm, and the optimum height and width of the silicon strip waveguide were 400 nm and 400 nm, respectively. The six-level spectroscopic model was presented, and the gain characteristics of the slot waveguide amplifier were numerically simulated. A net gain of 8.2 dB was achieved, which provided new ideas and directions for waveguide amplifiers.

## 1. Introduction

Research of integrated optics has developed rapidly [1,2,3]. The diverse passive and active nanophotonic devices based on silicon-on-insulator (SOI) waveguides and silicon nitride (Si_3_N_4_) waveguides such as modulators [4,5], filters [6,7], wavelength division multiplexers [8,9], and detectors [10,11] have been widely developed and have shown numerous applications in many fields. However, challenges still exist. Loss of the large-scale on-chip integrated devices limits the device performance and increases the bit error rate of the signal, which hinders the development of large-scale integration [12,13]. On-chip erbium-doped waveguide optical amplifier (EDWA) can compensate for the loss effectively [14,15,16,17]. It is an efficient way to overcome this problem. Si and Si_3_N_4_ cannot efficiently perform the task of integrating optical interconnections alone. Therefore, it is highly necessary to integrate with other active materials. The novel slot waveguide, which can strongly confine the light in a narrow slot region of low refractive index material [18,19,20], is more likely to integrate with other materials than the traditional strip waveguide. The density of the light field in the slot is nearly 20 times higher than that of a conventional rectangular dielectric waveguide and will promote the signal and pump interaction with the gain medium. This kind of waveguide amplifier based on slots is of great value for improving the gain performance and reducing the threshold pump power. In recent years, few reports regarding the optical waveguide amplifiers based on slots integrated with erbium-doped materials have been reported. In 2012, the Er/Yb silicate slot waveguide amplifier was fabricated and achieved a signal 1.7 dB gain at 1.53 μm in a waveguide with a 6 mm long slot pumped at 1476 nm when the Er^3+^ concentration was confirmed to be 1 × 10^27^  m^−3^ [21]. However, the inorganic material is difficult to integrate with slots because of the complicated technology, expensive equipment, and long cycle. Erbium-doped polymer materials have the outstanding advantages of high bandwidth, low cost, species diversity, and ease of realizing large-scale and high-density integration [22,23,24,25]. Integrating with slots for EDWA fabrication is a good decision. In 2014, the potential for large optical gain improvement of erbium-doped rich polymers integrated into silicon-slot waveguides amplifier pumped at 1480 nm was shown [26]. In 2020, the gain characteristics of the hybrid slot waveguide amplifiers integrated with NaYF_4_:Er^3+^ NPs-PMMA pumped at 1480 nm were analyzed. A net gain of 5.78 dB was achieved with the length of 1.5 cm when the Er^3+^ concentration was 1.3 × 10^27^ m^−3^ [27]. In this case, the gain of the slot waveguide amplifier with the polymer as gain medium is still not ideal. This is mainly because Si has strong absorption of 980 nm light. Therefore, 1480 nm was chosen as a pump and pure erbium-doped polymer as gain medium. The low-pumping efficiency at 1480 nm and the low-Er^3+^-luminescence intensity of pure erbium-doped materials without Yb^3+^ sensitization limited the amplification properties which could not meet the requirements of optical communications. To overcome this issue, a Si_3_N_4_ platform with low loss and a weak absorption effect on 980 nm was employed [28,29]. A Si_3_N_4_ slot instead of slot-based Si confined the light field in the slot region tightly. Er^3+^ and Yb^3+^codoped polymer material was used as gain medium. 

In this work, the core-shell α-NaYF_4_/β-NaLuF_4_: Yb^3+^, Er^3+^ nanocrystals were synthesized using a mild hydrothermal method and then copolymerized with methyl methacrylate (MMA) to fabricate NaYF_4_/NaLuF_4_: Yb, Er NPs-PMMA as gain medium. The fluorescence emission intensity of the nanocrystals coated with the shell increased nearly six times compared to α-NaLuF_4_. The doped Er^3+^ concentrations had been increased an order compared with traditional physical doping methods. The Er^3+^ concentration doped was 2.8 × 10^26^ m^−3^. The slot waveguide was optimized by the semivector finite difference method considering the maximum confinement factors and the minimum effective mode area. The optimum width of the slot was 200 nm, and the optimal height and width of the silicon strip waveguide were 400 nm and 400 nm, respectively. The six-level spectroscopic model was presented, and the gain characteristics of the slot waveguide amplifier were numerically simulated. A net gain of 8.2 dB was when the transmission loss was 3 dB/cm. The gain was enhanced compared with the silicon slot waveguide amplifier even though the pure Er^3+^ concentration doped was nearly ten times higher than the Er^3+^ and Yb^3+^ codoped polymer material. It provided new ideas and directions for waveguide amplifiers.

## 2. Experiments Details

### 2.1. Preparation of NaYF_4_/NaLuF_4_: Yb, Er NPs-PMMA

The performance of medium gain material is an important factor affecting EDWA gain, particularly the Yb-Er ion doping concentration and the luminescence intensity. However, these two factors are often incompatible. On the one hand, small-sized nanoparticles tend to disperse easily in polymer matrix materials, but they have a large specific surface area and a significant surface luminescence quenching phenomenon, which in turn affects the luminescence intensity of nanoparticles. Larger-sized nanoparticles cause light scattering in the device, which leads to additional losses. On the other hand, nanoparticles are usually incorporated into polymer materials in a physically doped manner. Er^3+^ exists as inorganic salts and is poorly compatible with organic polymers, resulting in a low concentration of Er^3+^ optical activity in polymer matrix materials, in addition, the stability of the material also needs to be improved. Therefore, we carried out shell coating on the surface of rare-earth doped nanoparticles to improve the luminescence intensity of the nanoparticles and used the modification of unsaturated groups on the surface of nanoparticles to bond nanoparticles to polymer monomers through chemical bonds, which can greatly improve the incorporation of nanoparticles into polymers while solving the problems of easy agglomeration, low solubility, and poor stability of nanoparticles in polymers.

**Figure 1:**
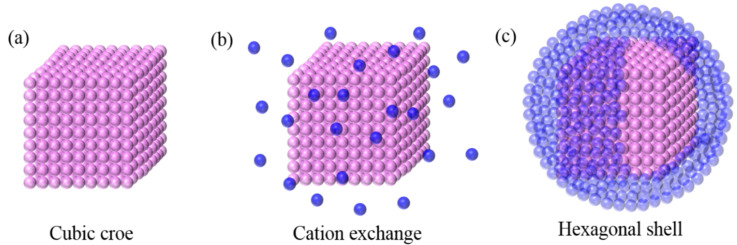
Schematic image of the heterogeneous induction procedure. (**a**) synthesis of cubic core; (**b**) Cation exchange; (**c**) Hexgonal shell coating.

The core-shell nanoparticles were synthesized using a mild hydrothermal method. In this condition, the unsaturated perssad on the surface of nanoparticles was protected by reacting at a relatively low temperature. The heterogeneous induction procedure was explored, as shown in Figure 1. The process is mainly divided into two major steps: first, was the preparation of the pure cubic phase of nuclear nanomaterial α-NaYF_4_. A total of 0.6 g of NaOH powder was dissolved in a solution of 20 mL oleic acid, 10 mL ethanol, and 10 mL deionized water and mixed by stirring to dissolve fully as a clear solution. A total of 0.5 mmol of YCl_3_·6H_2_O powder was weighed and dissolved in 4 mL of deionized water and stirred thoroughly to form a solution, and this solution was added dropwise to the above solution and stirred continuously until it was a homogeneous dissolution. A total of 2 mmol of KF·2H_2_O solid was dissolved in 4 mL of deionized water. It was stirred continuously until the solution was slowly dripped into the above-mixed solution and then stirred well to obtain a semi-white solution. This well-mixed solution was divided equally into two polytetrafluoroethylene-lined high-pressure reactors, heated at 160 °C for 240 min, and cooled to room temperature to obtain a mixed solution containing α-NaYF4 nanoparticles. Then, the shell growth link was performed. A total of 0.3 g NaOH powder was weighed and dissolved in 10 mL oleic acid, 5 mL ethanol, and 1 mL deionized water to prepare a solution. A 20 mL solution containing α-NaYF_4_ nuclear nanoparticles was mixed with the above solution and stirred. A total of 0.195 mmol of LuCl_3_·6H_2_O powder, 0.05 mmol of YbCl_3_·6H_2_O powder, and 0.005 mmol of ErCl_3_·6H_2_O solid were weighted and dissolved in 2 mL of deionized water. A mixture of nuclear nanoparticles was slowly dropped and stirred continuously to form a homogeneous solution. A total of 2 mmol of KF·2H_2_O was dissolved in 2 mL of deionized water and stirred vigorously and continuously to obtain the mixture. Then it was dispensed into two polytetrafluoroethylene-lined high-pressure reactors and heated at 160 °C for 480 min for the second-step crystal-growth process, during which cation exchange occurred between the newly added Lu^3+^ and Y^3+^. A NaY_x_Lu_(1-x)_F_4_ heterointerface was formed on the nuclear surface. Lattice mismatches of NaYF_4_ and NaLuF_4_ could provide additional driving forces to trigger the cubic-to-hexagonal phase transition. At the end of growth, the mixture was slowly dropped to room temperature, centrifuged, and vacuum-dried to obtain a white powder, core-shell structure NaYF_4_/NaLuF_4_: 20%Yb^3+^, and 2%Er^3+^ nanoparticles.

### 2.2. Characterization of NaYF_4_/NaLuF_4_: Yb, Er NPs-PMMA

**Figure 2:**
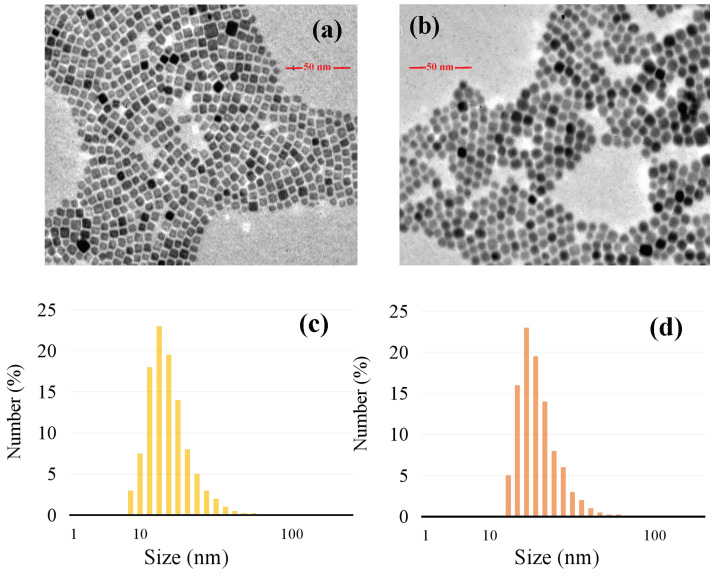
(**a**) TEM images of NaYF_4_ core; (**b**) TEM images of core-shell NaYF_4_/NaLuF_4_: Yb^3+^, Er^3+^; (**c**) Size distribution of NaYF_4_ core; (**d**) Size distribution of core-shell NaYF_4_/NaLuF_4_: Yb^3+^, Er^3+^.

A spot of the nanoparticles powder was dissolved in cyclohexane to form a transparent solution and dropped onto a carbon film copper mesh. The morphology and size of the nanoparticles were observed with Hitachi H-600 transmission electron microscopy. The TEM images of the nuclear α-NaYF_4_ material are presented in Figure 2a. The nanoparticles with a regular square shape were dispersed uniformly. The particle size we measured was within the range of 10–15 nm, as shown in Figure 2c. The average particle size of nuclear NaYF_4_ nanoparticles was around 13 nm, according to Scheller’s formula. TEM images of the nanoparticles coated shell are shown in Figure 2b. The morphology of the nanoparticles appeared spherical, which was because the size of the hexagon tended to be spherical when it was small. They were dispersed uniformly without agglomeration. Compared with uncoated particles, the size of the particle was increased; as shown in Figure 2d, the size of the nanoparticles after shell coating was approximately 21 nm.

**Figure 3:**
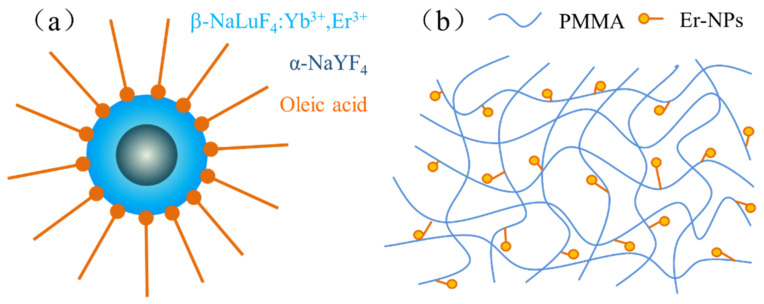
(**a**) Schematic images of core-shell NaYF_4_/NaLuF_4_: Yb^3+^, Er^3+^; (**b**) Schematic images of nanoparticles copolymerized with MMA.

The nanoparticles we synthesized were coated with oleic acid. The C=C, C=O double bonds from oleic acid ligands were protected well using the solvothermal method. Nanoparticles were chemically bonded to the polymer monomer using unsaturated ligand double bonds, and a schematic image of the covalent linking nanocomposites is shown in Figure 3. We dissolved 0.1 mmol nanoparticles in 4 mL butyl acetate. A total of 10 g methyl methacrylate (MMA) was weighed and dissolved in 2 mL butyl acetate. Then, polymerization initiator azobisisobutyronitrile (ABIN) was added together in a single-port flask. We put the flask in an oil bath followed by heating (65 °C) and stirring for 60 min to complete the prepolymerization reaction. The solution containing nanoparticles was added dropwise into the flask. We increased the temperature of the oil bath (80 °C) and continuously stirred it to achieve the copolymerization reaction. The NPs-PMMA covalent linking nanocomposites were obtained by free-radical polymerization, ionic polymerization, and the polymerization of nanoparticles with organic polymer monomers under the catalyzation of free-radical initiators and ionic-type initiators. The doped Er^3+^ concentration was enhanced to 2.8 × 10^26^ m^−3^, which was increased compared to traditional physical doping.

**Figure 4:**
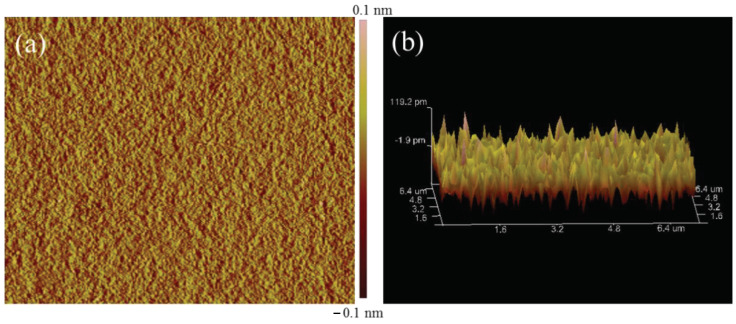

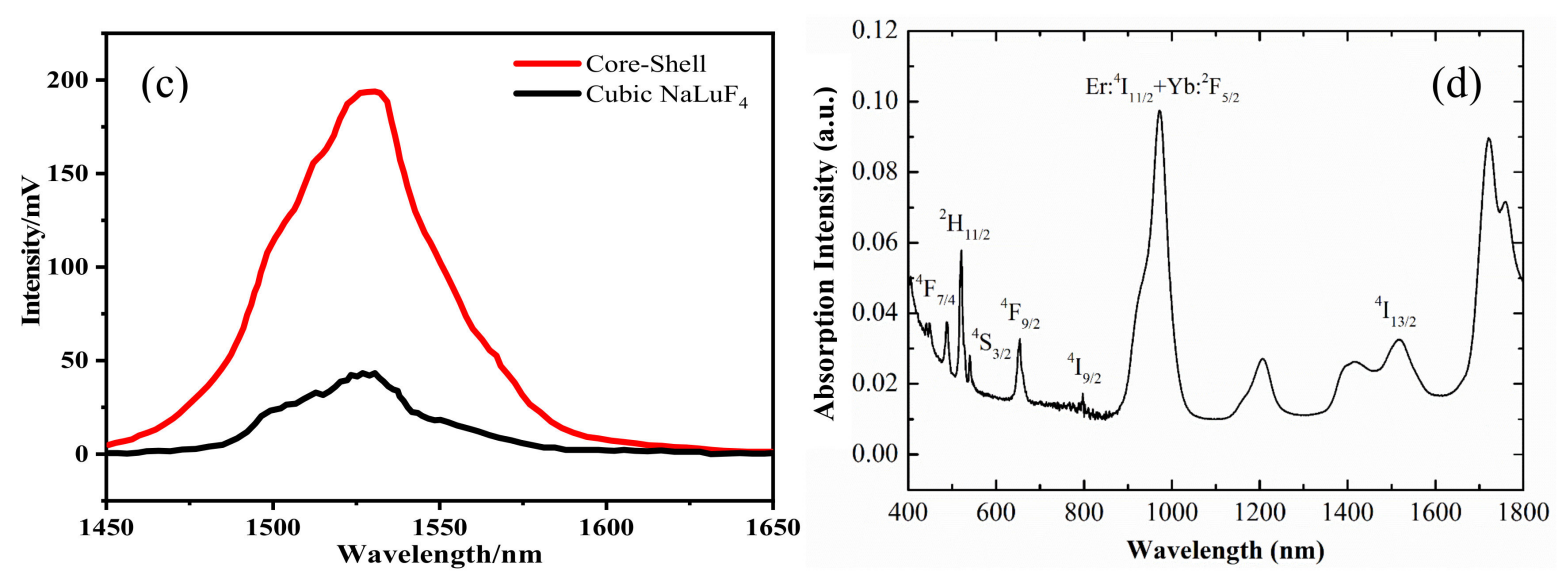
(**a**) AFM 2D images and (**b**) AFM 3D images of the NaYF_4_/NaLuF_4_: Yb, Er NPs-PMMA; (**c**) Emission spectrums of NaYF_4_/NaLuF_4_: Yb, Er NPs and NaLuF_4_: Yb, Er NPs under 980 nm excitation; (**d**) Absorption spectrum of NaYF_4_/NaLuF_4_: Yb, Er NPs-PMMA.

The critical properties of film forming, emission spectra, and absorption spectra of the gain medium were characterized and were found to impact the gain performances of EDWA. The NPs-PMMA was spin-coated on Si and heated by 120 °C for 2 h in the oven to form a 7 μm-thick film. Atomic force microscopy (AFM) was used to characterize the film-forming properties of the material shown in Figure 4a,b. As shown, the film surface was flat and smooth without nanoparticle precipitation. The surface roughness of the film was measured to be only 0.349 nm (scan area 20 μm × 20 μm), which was an order of magnitude lower than traditional physical doping. This suggests that it is an ideal material for preparing waveguide devices. The infrared emission spectrums pumped by 980 nm of the α-NaYF_4_/β-NaLuF_4_: Er^3+^, Yb^3+^ NPs, and α-NaLuF_4_ NPs with the same doping concentration were measured. As illustrated in Figure 4c, the main emission peak of both materials was at 1530 nm, corresponding to the ^4^I_13/_2→^4^I_15/2_ energy level transition of Er^3+^. Nanoparticles coated with a shell had a stronger emission than cubic-phase NaLuF_4_ of the same size. The fluorescence performance was significantly improved by nearly six times. The full width at half-maximum (FWHM) of the NaYF_4_/NaLuF_4_: Er, Yb NPs fluorescence spectrum was broadened to 62 nm. The absorption spectra of the core film were characterized by a Cary500 Scan UV-Vis-NIR spectrophotometer, as shown in Figure 4d. The typical transitions of ground-state Er^3+^ to each excited-state energy level corresponded to the absorption peaks in the figure. Yb^3+^ was codoped in, and the absorption peak at 976 nm was much stronger than that of pure doped Er^3+^, which facilitated the absorption and improved the efficiency rate of pump light.

### 2.3. Optimization of the Slot Waveguide

**Figure 5:**
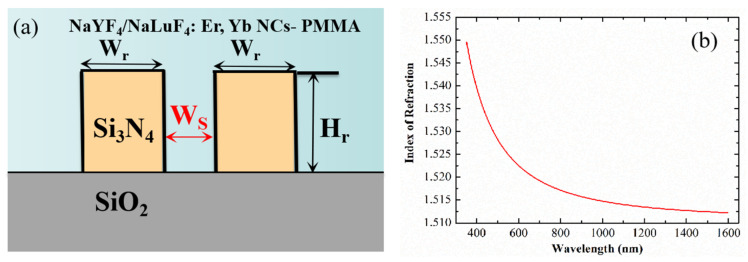
(**a**) Schematic of slot waveguide; (**b**) The measured refractive index curve of core material.

The electric field intensity in the vertical medium interface at the sidewall will be modified by the factor of $\frac{n_{Si_{3}N_{4}}^{2}}{n_{NCs-PMMA}^{2}}$ because of the electric displacement continuity boundary condition (*D* *=* *εE*) for the horizontal dielectric. Then, the light can be confined within the nanoscale low-refractive index slot, contributing to the high light intensity. A schematic image of the slot waveguide is shown in Figure 5a. Taking advantage of the high electric field intensity, the interaction between the signal light, pump light, and gain medium will be promoted. The stronger the ability of the slot waveguide to limit the light field, the stronger the interaction with the gain medium, and greater gain can be achieved. Meanwhile, a smaller effective cross-sectional area of the slot waveguide can facilitate a reduction in the threshold power of EDWA. Therefore, we optimized the size of the slot waveguide in terms of both the ability to confine the light and the effective cross-sectional area. We filled the NaYF_4_/β-NaLuF_4_: Er, Yb NCs-PMMA into the slot as gain medium and measured its refractive index with an ellipsometer, as shown in Figure 5b. The measured values of the core layer were 1.512 at 1530 nm and 1.52 at 980 nm, respectively. 

**Figure 6:**
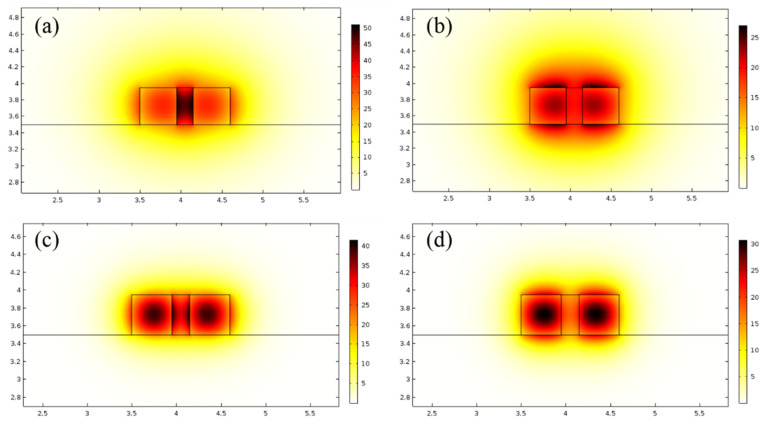
The modal profiles of Si_3_N_4_ slot waveguide (**a**) TE polarization at 1530 nm; (**b**) TM polarization at 1530 nm; (**c**) TE polarization at 980 nm; (**d**) TM polarization at 980 nm.

The finite element method (FEM) was used to analyze the modal profiles of the Si_3_N_4_ slot waveguide with a height (H_r_) of 450 nm, width (W_r_) of 450 nm, and slot width (W_s_) of 200 nm. The optical field distributions under TE and TM polarization at 1530 nm are shown in Figure 6a,b, respectively. The optical field distributions under TE and TM polarization at 980 nm are shown in Figure 6c,d respectively.

Under TE polarization, the slot can confine the light well, while more light fields leak into the upper and lower cladding under TM polarization. This was not conducive to the interaction between the light field and the gain polymer. Therefore, we only considered the TE polarization mode, and the related parameters under the TM polarization mode in this paper were only used for reference and comparison.

#### 2.3.1. Optimization of Slot Waveguides Combined with Maximum Confinement Factors

For a more intuitive view of the slot’s ability to restrict the light, we defined the overlapping integration factor Γ_S_ of the slot waveguide to represent the effective utilization of energy, as shown in Equation (1), so we searched for a maximum in all the considered cases, which could be used to achieve higher gain [30]. The finite difference method (FDM) was used to calculate Γ_S_ to primarily optimize the height of Si_3_N_4_ (H_r_), the width of Si_3_N_4_ (W_r_), and the slit width (W_S_).

(1) $$\Gamma_{S}=\frac{\iint_{S}{\left|E(x,y)\right|}^{2}dxdy}{\iint_{Total}{\left|E(x,y)\right|}^{2}dxdy}$$

**Figure 7:**
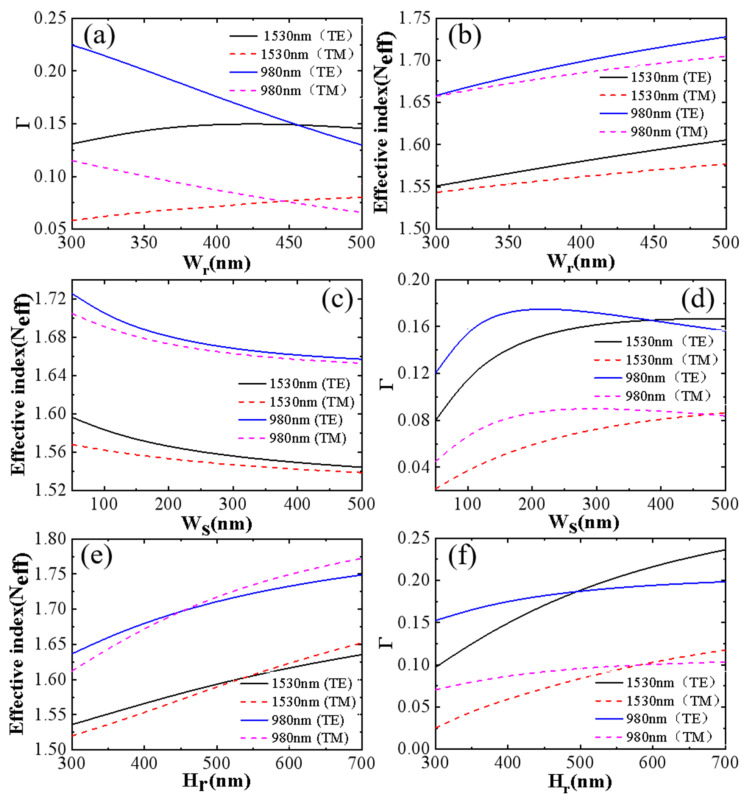
(**a**) N_eff_ as a function of Wr (W_S_ = 200 nm, H_r_ = 400 nm); (**b**) Γ_S_ as a function of W_r_ (W_S_ = 200 nm, H_r_ = 400 nm); (**c**) N_eff_ as a function of W_S_ (W_r_ = 400 nm, H_r_ = 400 nm); (**d**) Γ_S_ as a function of W_S_ (W_r_ = 400 nm, H_r_ = 400 nm); (**e**) N_eff_ as a function of H_r_ (W_r_ = 400 nm, W_S_ = 200 nm); (**f**) Γ_S_ as a function of H_r_ (W_r_ = 400 nm, W_S_ = 200 nm) under TE and TM polarizations at 1530 nm and 980 nm.

First, the effective refractive index (N_eff_) and Γ_S_ were calculated as a function of the width of W_r_ at 1530 nm and 980 nm under TE and TM polarizations shown in Figure 7a,b. The wavelength interval between the 980 nm pump light and signal light was quite large; the optical field distribution was significantly different. Then, the N_eff_ and Γ_S_ of the waveguide at signal wavelengths were quite different from those of pump wavelengths. Under the TE polarization mode, the light field was confined in the slot well. With increasing W_r_, N_eff_ also increased, and Γ_S_ at 980 nm gradually decreased. At the same time, ΓS at 1530 nm tended to saturate when W_r_ increased to 400 nm. This is due to the low impact of W_r_ at 1530 nm on the waveguide light field. At 980 nm, the light field was mostly confined to the Si_3_N_4_ strip waveguide. As Wr increased, more light fields were confined to the Si_3_N_4_ strip waveguide. As a result, N_eff_ increased while Γ_S_ gradually decreased. Next, we optimized WS combined with the N_eff_ and Γ_S_ curves at the signal and pump wavelengths under TE and TM polarizations, as revealed in Figure 7c,d. The curves under TE polarization tend to be basically consistent with that of TM polarization. When W_S_ increased, N_eff_ decreased. Additionally, Γ_S_ at a 980 nm wavelength decreased rapidly, increasing W_S_. It increased to extreme values and then decreased rapidly. Γ_S_ increased rapidly at 1530 nm as W_S_ increased and tended to be saturated. Maximum Γ_S_ was obtained when W_S_ was 200 nm. This is because with increased W_S_, light field leakage to the upper and lower cladding was gradually controlled, and more light fields were included in the slot. Therefore, Γ_S_ increases rapidly at this time, accompanied by a rapid decrease in N_eff_. However, with increased W_S_, the coupling resonance of the abrupt light field at the interface between slot dielectrics was weakened. Thus, the confinement of the light field in the slot was impaired, and some light fields leaked to the cladding. As a result, N_eff_ and Γ_S_ gradually decreased, and the rate of decrease in N_eff_ became slower. Combined with the dependence of N_eff_ and Γ_S_ on the different widths of H_r_ shown in Figure 7e,f, we optimized the H_r_ of the waveguide. H_r_ is the main factor affecting Γ_S_. For any waveguide slot size, increasing its Hr will make Γ_S_ increase. As H_r_ increased, both N_eff_ and Γ_S_ increased monotonically. Since the light at 980 nm already confined most of the light to the Si_3_N_4_ strip waveguide, it continued to increase its height and had little effect on Γ_S_. Meanwhile, too high an H_r_ led to an increase in transmission loss and introduced extra difficulty to the coupling between the optical fiber and the end face of the waveguide.

#### 2.3.2. Optimization of Slot Waveguide Combined with Minimum Effective Mode Area

We combined the effective cross-sectional area (*A_eff_*) to further optimize the size of the slot. The definition formula for *A_eff_* is shown in Equation (2) [27]:

(2) $$A_{eff}=\frac{(\iint {\left|E(x,y)\right|}^{2}dxdy{)}^{2}}{\iint {\left|E(x,y)\right|}^{4}dxdy}$$

**Figure 8:**
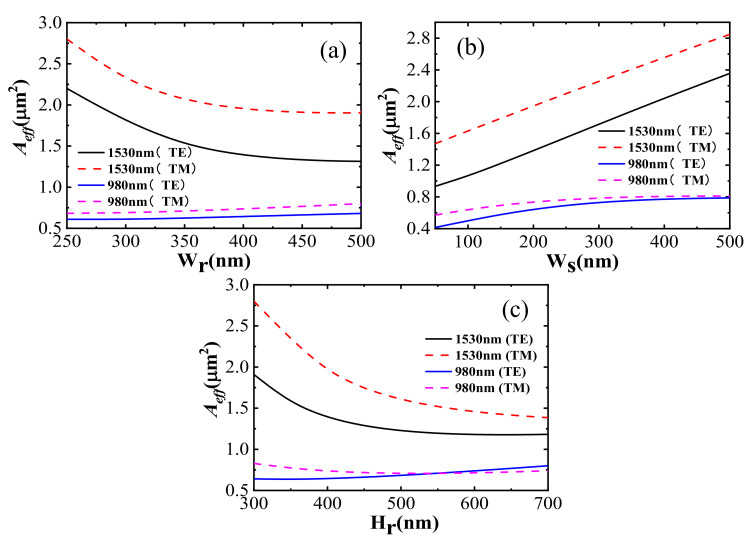
Under TE and TM polarizations at 1530 nm and 980 nm (**a**) dependence of *A_eff_* on W_r_ (W_S_ = 200 nm, H_r_ = 400 nm); (**b**) dependence of *A_eff_* on W_S_ (W_S_ = 200 nm, H_r_ = 400 nm); (**c**) dependence of *A_eff_* on H_r_ (W_r_ = 400 nm, H_r_ = 400 nm).

The higher the Γ_S_ value, the higher the obtained gain. The smaller the *A_eff_*, the smaller the threshold pump power. As shown in Figure 8, we calculated *A_eff_* for different slot sizes. The *A_eff_* under TE polarization at the same wavelength was much smaller than the *A_eff_* under TM polarization. Thus, only the TE polarization mode was analyzed in this paper. As shown in Figure 8a, with increasing W_r_, *A_eff_* first decreased quickly at the wavelength 1530 nm. When W_r_ reached 400 nm, *A_eff_* tended to saturate. *A_eff_* remained almost unchanged as W_r_ increased at 980 nm. Combined with the joint analysis of Figure 7a,b, 400 nm was demonstrated to be the optimal value for W_r_. Different W_S_ and *A_eff_* relationship curves under TE and TM polarizations at 1530 nm and 980 nm are shown in Figure 8b for when H_r_ was 400 nm and W_r_ was 400 nm. *A_eff_* slowly increased. As W_S_ increased from 50 nm to 500 nm at 980 nm, *A_eff_* increased by less than 0.1 μm^2^. Thus, *A_eff_* at 1530 nm was mainly considered along with the increase in W_S_. *A_eff_* curves trended along linear growth. Combined with the joint analysis of Figure 7c,d, 200 nm was demonstrated to be the optimal value for W_S_. *A_eff_* with different H_r_ under TE and TM polarization at 1530 nm and 980 nm wavelengths were simulated in Figure 8c for when W_r_ was 400 nm and W_S_ was 200 nm. *A_eff_* slowly increased with an increase in H_r_ at the pump wavelength. *A_eff_* first decreased rapidly to an extreme value and then slowly increased with the increase in H_r_ at a signal wavelength. Combined with the joint analysis of Figure 7e,f, we believe that 400 nm is the best-optimized value for H_r_. In this condition, under TE polarization, Γ_S_ is 0.1500 and 0.1753, and *A_eff_* is 1.3829 µm^2^ and 0.6436 µm^2^ in the slit region at 1530 nm and 980 nm wavelengths, respectively.

## 3. Results

**Figure 9:**
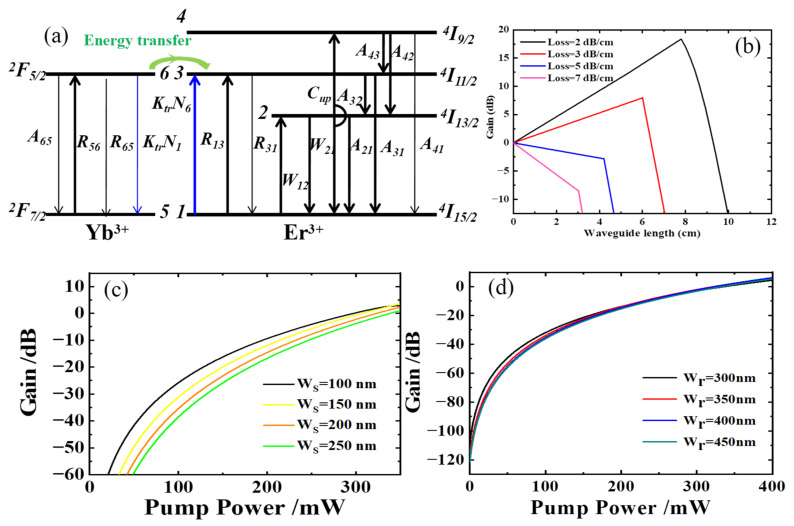
(**a**) Energy level transitions for Er^3+^-Yb^3+^ codoped systems; (**b**) The gain versus coordinate pump power at 980 nm for different waveguide lengths; (**c**) The gain versus coordinate pump power at 980 nm for different W_S_; (**d**) The gain versus coordinate pump power at 980 nm for different W_r_.

The novel Er and Yb codoped waveguide amplifiers were presented by integrating NaYF_4_/NaLuF_4_: Er^3+^, Yb^3+^ NCs-PMMA with a Si_3_N_4_ slot waveguide. To analyze the gain performance of the device accurately, we established a transition model of an Er-Yb codoped six-level system pumped at 980 nm, as shown in Figure 9a. The Yb^3+^ ions in the ground state ^2^F_7/2_ were excited by a 980 nm pump absorbing the energy and then transitioned upward to level ^2^F_5/2_. Because ^2^F_5/2_ and ^2^F_7/2_ levels of Yb^3+^ had the same energy spacing between Er^3+ 4^I_11/2_ and ^4^I_15/2_ levels, and erbium ytterbium atoms were very far apart, Yb^3+^ ions rapidly transmitted energy to Er^3+^ ions in the ground state by cross-relaxation. Thus, they transited from the ^4^I_15/2_ level to the excited-state level ^4^I_11/2_. Due to the instability of ^4^I_15/2_, Er^3+^ ions quickly transferred to the metastable state ^4^I_13/2_ through the nonradiative relaxation to form population inversion. Then, they transitioned down to the ground state ^4^I_15/2_ by excited emission and emitted photons with the same frequency as the signal light. The amplification of the signal light was realized. To build an accurate simulation model, we also considered cooperative up-conversion C_up_ between metastable levels ^4^I_13/2_ and ^4^I_9/2_, ^4^I_15/2_. Spontaneous radiation transitions from ^4^I_9/2_, ^4^I_11/2_, and ^4^I_13/2_ to their next levels were taken into account, as well as ^2^F_5/2_^Yb^ to ^2^F_7/2_^Yb^. Amplified spontaneous emission (ASE) was neglected. A six-level spectroscopic model pumped at 980 nm was presented. The rate equations and propagation equations were solved. Incorporated with the characteristic parameters of the gain medium, the gain G at any point of the amplifier in the transmission direction z was calculated by the fourth-order Runge–Kutta method. G can be expressed in dB as:

(3) $$G=10\mathrm{lg}\frac{P_{S}(z)}{P_{S}(0)}$$

where *P_S_*(0) is the input signal power and *P_S_*(*z*) is the output signal power. As illustrated in Equation (3), a higher gain can be achieved with small input signal power. In combination with experimental experience, a 0.1 mW input power was selected. The Si_3_N_4_ slot waveguide amplifier was fabricated via the following steps: first, the Si_3_N_4_ film on SiO_2_ was first annealed at a high temperature of 1050–1150 °C for 3–7 h to remove the hydrogen bonds on the surface and inhibit the formation of O-H and N-H bonds before preparing the waveguide. The positive electron resistance ZEP520 with a thickness of 400 nm was spin-coated on the Si_3_N_4_ wafer. Then, the standard pattern exposure was performed using an electron beam. After image development and ICP (SPTS) etching for 140 s, the S_i3_N_4_ slot waveguide was obtained. Finally, a 3 µm-thick PMMA film was spin-coated onto the S_i3_N_4_ slot as the core layer and then baked at 120 °C for 2 h. The S_i3_N_4_ slot waveguide amplifier was obtained. We simulated the gain versus coordinate waveguide length with different transmission losses in Figure 9b. The optimal waveguide length corresponding to different transmission losses was different. The corresponding optimal waveguide length was longer when the transmission loss was smaller. When the transmission loss was only 2 dB/cm, the optimal waveguide length was approximately 8 cm, and a net gain of nearly 20 dB could be obtained. As transmission losses gradually increased, the gain values gradually decreased. No net gain was generated when the transmission loss was 5 dB/cm. This is mainly due to the small refractive index difference between Si_3_N_4_ and gain polymer materials, thus resulting in a smaller overlapping integration factor than the SOI slot waveguide. While the main advantage of preparing EYCDWA using a Si_3_N_4_ slot waveguide was the small transmission loss of the waveguide, a long waveguide could be used to improve the gain. At present, the minimum loss of the Si_3_N_4_ slot waveguide measured was approximately 3 dB/cm, the optimized length of the device was 6 cm, and the net gain obtained was approximately 8.2 dB. The doped Er^3+^ concentration will be further improved, and the optimum length of the waveguide will be shortened. Under the optimized conditions above, we simulated the gain characteristics of Si_3_N_4_ slot waveguide amplifiers with different slot widths, as shown in Figure 9c. The figure shows that the smaller the slot width, the smaller the pump threshold power is, which was associated with the A_eff_ of the waveguide. When the pump power was less than 300 mW, the gain increased with W_S_. Additionally, the gain values corresponding to each slot width were nearly overlapped as the pump power continued to increase. Therefore, the optimized range of W_S_ was relatively larger. In this paper, considering polymer packing and transmission loss, the W_S_ with 200 nm was selected as the optimized width. Figure 9d shows the gain versus pump power for different W_r_s. Within this width range, the gain curves of each device almost completely coincided, mainly due to W_r_ not having direct effects on the light field. Within the W_r_ range of 300 nm to 450 nm, the gain presented minor variations. This feature provides convenient conditions for preparing devices and allows certain process tolerances.

## 4. Conclusions

In summary, we synthesized α-NaYF_4_/β-NaLuF_4_: Yb^3+^, Er^3+^ nanoparticles coated with oleic acid using a mild hydrothermal method, whose luminescence intensity was improved at 1.53 µm nearly six-fold. Then, the nanoparticles were copolymerized with methyl methacrylate (MMA) to fabricate NaYF_4_/NaLuF_4_: Yb, Er NPs-PMMA. The doped Er^3+^ concentration was 2.8 × 10^26^ m^−3^. NaYF_4_/NaLuF_4_: Yb, Er NPs-PMMA was filled into a Si_3_N_4_ slot waveguide as a core to promote the interaction between the light field and the Er-Yb codoped gain medium, and 980 nm was used as a pump light to improve the gain performance. The optimum size of the Si_3_N_4_ slot waveguide with an H_r_ of 400 nm, W_S_ of 200 nm, and Wr of 400 nm were obtained by combining the confining factor and effective cross-sectional area via the finite difference method. A six-level system model of the Yb-Er codoped system was established, and the gain characteristics were simulated. Theoretically, when transmission loss was 3 dB/cm, a net gain of 8.2 dB could be obtained. With further optimization of loss and doped Er^3+^ concentration, ultrahigh gain can be envisaged.

## Data Availability

Not applicable.
